# Integrated Analysis of Molybdenum Nutrition and Nitrate Metabolism in Strawberry

**DOI:** 10.3389/fpls.2020.01117

**Published:** 2020-07-28

**Authors:** Li Liu, Hongmei Shi, Shaoxuan Li, Mingyue Sun, Rui Zhang, Yongmei Wang, Fengshan Ren

**Affiliations:** ^1^ Shandong Academy of Grape, Shandong Academy of Agricultural Sciences, Jinan, China; ^2^ Fruit & Tea Institute, Qingdao Academy of Agricultural Sciences, Qingdao, China; ^3^ College of Horticulture Science and Engineering, Shandong Agricultural University, Taian, China

**Keywords:** molybdenum, nitrate, molybdenum deficiency, strawberry, ^15^NO_3_^-^ use efficiency

## Abstract

Molybdenum (Mo) is a component of the Mo cofactor (Moco) of nitrate reductase (NR) and is therefore essential for nitrate metabolism. However, little is known about Mo deficiency phenotypes or about how physiological and molecular mechanisms of Mo uptake and transport influence nitrate uptake and utilization in strawberry. Here, we used physiological and cytological techniques to identify Mo deficiency phenotypes in strawberry. Seedlings cultured with MoO_4_^2-^ grew well and exhibited normal microstructure and ultrastructure of leaves and roots. By contrast, seedlings cultivated under Mo-deficient conditions showed yellow leaf blades and ultrastructural changes such as irregular chloroplasts and unclear membrane structures that were similar to the symptoms of nitrogen deficiency. We cloned and analyzed a putative molybdate transporter, *FaMOT1*, which may encode a molybdate transporter involved in the uptake and translocation of molybdate. Interestingly, the addition of the molybdate analog tungstate led to lower tissue Mo concentrations, reduced the translocation of Mo from roots to shoots, and increased the plants’ sensitivity to Mo deficiency. Seedlings cultivated with MoO_4_^2-^ altered expression of genes in Moco biosynthesis. As expected, NR activity was higher under sufficient MoO_4_^2-^ levels. Furthermore, seedlings grown on Mo-deficient medium exhibited decreased ^15^NO_3_^-^ translocation and lower ^15^NO_3_^-^ use efficiency. These findings represent an important step towards understanding how molybdate transport, concentration, and deficiency symptoms influence nitrate uptake and utilization in strawberry.

## Introduction

As one of the most popular cultivated fruits in the world, strawberry (*Fragaria* × *ananassa* Duchesne) offers not only pleasant flavors but also abundant mineral nutrients such as molybdenum (Mo), a transitional element essential for nearly all living organisms (Arnon and Stout, 1939; Aharoni et al., 2004; Tejada-Jimenez et al., 2009; Akhatou and Recamales, 2014). In humans, Mo is required for the biosynthesis of the Mo cofactor (Moco). Molybdenum cofactor deficiency (MoCD) is a severe autosomal recessive inborn error in metabolism (Atwal and Scaglia, 2016) that leads to the loss of activities of the molybdenum-cofactor-dependent enzymes such as sulfite oxidase, xanthine dehydrogenase, aldehyde oxidase, and the mitochondrial amidoxime reducing component (Tejada-Jiménez et al., 2013). These enzymes have essential roles in vital processes such as purine metabolism and sulfite detoxification. Their deficiency causes accumulation of sulfite, taurine, S-sulfocysteine, and thiosulfate and contributes to severe neurodegeneration in newborns and early childhood death (Mendel and Schwarz, 2011). The incidence of MoCD in humans is less than one in 100,000. However, in plants, Mo deficiency occurs frequently in acidic soils, well-drained soils, and soils rich in adsorbing oxides such as iron (Fe) oxides (Kaiser et al., 2005; Marschner and Rengel, 2012). Worldwide, approximately 70% of arable land is characterized as acidic, and in China, up to four million hectares of arable land suffer from Mo deficiency (von Uexküll and Mutert, 1995; Gao et al., 2016). Therefore, Mo deficiency is a widespread problem for which solutions are urgently needed.

Adequate Mo is indispensable for the binding to Mo-containing enzymes of plant nitrate assimilation, purine metabolism, phytohormone production, and sulfite detoxification (Arnon and Stout, 1939; Tejada-Jiménez et al., 2013; Mendel and Leimkühler, 2015). MoO_4_^2-^ is the main form in which plants absorb and utilize Mo, and they may experience Mo deficiency on acidic soils because Mo availability decreases when MoO_4_^2-^ forms sediments by binding with free metallic elements such as Fe, Mg, and Al (Gong et al., 1998). Common phenotypes of Mo deficiency in crops and vegetables include stunted growth, yellow leaves, and the characteristic “whiptail” symptoms such as rolling, curling, and scorching of leaf margins, mottled leaf lesions, and marginal leaf necrosis (Arnon and Stout, 1939). Excessive use of nitrogen (N) and phosphate (P), together with insufficient application of organic fertilizer and trace elements, have frequently resulted in orchard soil acidification in China. Because there is limited information on Mo deficiency in fruit crops, it is urgent to characterize Mo deficiency phenotypes and to elucidate the physiological and molecular mechanisms of Mo uptake and transport, thereby helping to improve fruit yield and quality.

In prokaryotes, Mo is taken up and transported by high-affinity ModABC transporters that belong to the ATP-binding cassette protein superfamily (Wanner and Soppa, 1999; Zahalak et al., 2004). In eukaryotes, two types of Mo transporters have been found in the green alga *Chlamydomonas reinhardtii*: one with high affinity and low capacity, and the other with low affinity and high capacity (Tejada-Jiménez et al., 2007; Tejada-Jiménez et al., 2011). In *Arabidopsis thaliana*, the sulfur transporter (SULTR) family members SULTR5.1 and SULTR5.2 were shown to be Mo-specific transporters and named AtMOT1;2 and AtMOT1;1 (Tomatsu et al., 2007; Gasber et al., 2011; Tejada-Jiménez et al., 2013). In *Lotus japonicas*, LjMOT1 has been shown to be an essential high-affinity molybdate transporter (Duan et al., 2017), and in *Oryza sativa*, quantitative trait locus (QTL) analysis has shown that OsMOT1;1 is a molybdate transporter (Huang et al., 2018).

The *C. reinhardtii* antisense mutant *mot1* exhibited lower *MOT1* expression and reduced MoO_4_^2-^ transport (Tejada-Jiménez et al., 2007). NR activity was also lower in *mot1*, presumably as a result of Mo deficiency. Likewise, *atmot1;2* mutant seedlings showed reduced nitrate levels and slightly lower NR activity compared with the wild-type plants (Gasber et al., 2011). However, in the absence of MoO_4_^2-^, the *atmot1;2* mutant showed substantially higher nitrate levels and significantly lower NR activity (Gasber et al., 2011). In *Lotus japonicas*, changes in NR activity coincided with changes in Mo concentration, which strongly suggested that Mo is an essential part of NR and plays a vital role in NR catalysis. In *Cucumis sativus*, the model of the molecular interactions connecting Mo and Fe homeostasis was firstly proposed by Vigani et al. (2016). To date, molybdate transporters that control Mo concentration and influence the reduction, assimilation, and utilization of nitrate have not been characterized in fruits.

Currently, relatively little is known about Mo deficiency phenotypes or about Mo transporters and their molecular mechanisms in fruits such as strawberry (*Fragaria* × *ananassa* Duchesne). Such information would help to facilitate the development of horticultural varieties with enriched Mo concentrations and improved Mo nutrition in Mo-deficient soils. In this research, we identify Mo deficiency phenotypes in strawberry using physiological and cytological techniques. We show that *FaMOT1*probably encodes a molybdate transporter and that the addition of the molybdate analog anion tungstate resulted in lower Mo concentrations, reduced Mo translocation from roots to shoots, lowered the expression of genes in the Moco biosynthetic pathway, and greater sensitivity to Mo deficiency. We further demonstrate that Mo deficiency reduces NO_3_^-^ translocation and NO_3_^-^ use efficiency. Our findings permit a better understanding of Mo deficiency phenotypes and of how *FaMOT1* and Mo concentrations enhance nitrate translocation and utilization through the Moco biosynthetic pathway.

## Materials and Methods

### Plant Materials and Growth Conditions

Strawberry (*Fragaria* × *ananassa* Duch. cv. Akihime) stem tips from healthy tissue culture seedlings cultivated from stem tips were selected and inoculated on MS solid media containing (1) CK (−Na_2_MoO_4_, −Na_2_WO_4_, no Mo and W added to the media), (2) Mo treatment (+Na_2_MoO_4_, 0.25 mg/L), (3) W treatment (+Na_2_WO_4_, 0.25 mg/L), or (4) Mo+W treatment (+Na_2_MoO_4_, 0.25 mg/L +Na_2_WO_4_, 0.25 mg/L). One hundred and fifty replicate seedlings were grown under each condition. The seedlings were grown in a tissue culture room at 22°C under a 16/8 h light/dark cycle with a light intensity of 100–150 µmol m^−2^ s^−1^. Mo concentration **(**50 replicate seedlings for each treatment, each treatment contains three independent biological replicates**),** enzyme activities (50 replicate seedlings for each treatment, each treatment contains three independent biological replicates), and gene expression levels of the seedlings (10 replicate seedlings for each treatment, each treatment contains three independent biological replicates) were measured after 45 days of seedling culture. To analyze seedling microstructure (10 replicate seedlings for each treatment, each treatment contains three independent biological replicates) and ultrastructure (10 replicate seedlings for each treatment, each treatment contains three independent biological replicates), the samples were also obtained after 45 days of culture.

### Analysis of Mo Concentration

Mo concentrations were measured using the NexION™ 300 ICP-MS System (PerkinElmer, Waltham, MA, USA) following the methods of Filipiak-Szok et al. (2014). Roots and shoots were excised and washed with 0.5 mM calcium chloride solution three times, rinsed with Milli-Q water once, and dried overnight at 88°C. The samples (0.3 g per replicate) were digested with 5 mL of nitric acid (65% v/v) overnight, and then 2 mL of hydrogen peroxide (H_2_O_2_, 30% v/v) was added to dissolve the ashes. The final solution was transferred to a 50 mL volumetric flask with ultrapure water. Mo concentrations in the digested samples were determined using ICP-MS according to a previously published method (Liu et al., 2017). The results shown are the average of three independent biological replicates repeated three times.

### Measurements of Root Activities and Chlorophyll Content

Root activity was determined by TTC method. About 0.5 g of roots were placed in culture dishes with 10 ml equivalent mixture of 0.4%TTC solution and phosphate buffer in the dark for 1 h at 37°C. Then 2 ml of 1 mol/L sulfuric acid was added to stop the reaction. The roots were removed, dried and ground with 3 ~ 4ml ethyl acetate and a small amount of quartz sand to extract TTF. Transfer the red extract into the test tube with a small amount of ethyl acetate washing the residue for two to three times, and then transfer the residue into the test tube. Finally, add ethyl acetate to make the total amount of 10 ml, and the absorbance was recorded at 485 nm using a spectrophotometer.

The chlorophyll content was measured as described by Liu et al. (2017). About 0.2 g of strawberry leaves were placed in test tubes with 50 mL of 80% acetone and then incubated in the dark for 24 h at 4°C to extract the chlorophyll. The absorbance was recorded at 663 nm and 645 nm.

### Measurements of Root and Shoot Microstructure

Root tips and leaves were harvested from the strawberry seedlings after 45 days of treatment on the four MS media formulations described above. The strawberry leaves were cut into tissue blocks of about 2mm×2mm and the roots were cut into 2 cm pieces, which were fixed with FAA solution and then were stored at 4°C with 70% ethanol. The samples were dehydrated for 1h with 30, 50, 70, 80, 90, 95, 100, and 100% ethanol, respectively. After this, the samples were impregnated in 3/4 anhydrous ethanol-1/4 xylene, 1/2 anhydrous ethanol-1/2 xylene, 1/4 anhydrous ethanol-3/4 xylene, 100% xylene and 100% xylene, each step for 30min. The samples were subjected to endosmosis using paraffin. The sections were cut using the Leica hand slicer and stained with diaminobenzidine. The tissues were examined under an optical microscope (Olympus IX 71,Tokyo, Japan) as described in Lv et al. (2014).

### Measurements of Shoot Ultrastructure

Root tips and leaves were harvested from the strawberry seedlings after 45 days of treatment on the four MS media formulations described above. The strawberry leaves were cut into tissue blocks of about 2mm×2mm, which were put into a small glass bottle containing 2.5% glutaraldehyde solution (pH 7.5). They were settled at the bottom of the bottle for 24 h. All samples were fixed in 3.5% glutaraldehyde and washed with 0.1 M phosphate buffered saline (PBS).The samples were briefly post-fixed in 1% osmium tetroxide and dehydrated in an ascending ethanol series (10–70% ethanol). After this, the samples were subjected to endosmosis, and then imbedded and polymerized in Epon812 resin. Ultra-thin sections were cut using an LKB-V ultramicrotome and stained with uranium acetate and lead citrate. The tissues were then observed using a JEOL-1200EX TEM (JEOL, Tokyo, Japan) as described in Wu et al. (2015).

### Cloning the Full-Length *FaMOT1* cDNA

Using the accession numbers of *Arabidopsis thaliana* MOT1 family members, MOT1 protein sequences were obtained from the *A. thaliana* genome database (https://www.arabidopsis.org/). These were compared to the strawberry genome database (https://www.rosaceae.org/species/Fragaria/Fragaria×ananassa) to identify similar *MOT1* genetic sequences from strawberry. The most recent genome-wide strawberry data were downloaded from the GDR database (http://www.rosaceae.org/), and data for the MFS_MOT1 protein family (PF16983) were obtained from the Pfam database (http://pfam.xfam.org/). A HMMER 3.0 search was then performed to further verify MOT1-like sequences from the strawberry genome. Finally, a strawberry member of the MOT1 family was identified, and its gene was named *FaMOT1*.

A pair of degenerate primers, FaMOT1-F (5′- GTCGACATGGAGTCCCAAAACCC-3′) and FaMOT1-R (5′- GGATCCTCATGCATTCAAATCGTTAGAAT-3′) were designed based on the *FaMOT1* sequence with Primer 5.0. We then performed coding sequence (CDS) amplification of *FaMOT1* using Phusion High-Fidelity DNA Polymerase (Thermo Fisher Scientific) with strawberry cDNA as a template. The PCR products were collected using the TIANquick Midi Purification Kit (Tiangen Biotechnology, Beijing, China). After gel extraction, the PCR products were ligated to the vector from the pEASY-Blunt Zero Cloning Kit (TransGen Biotech) and used to transform *Escherichia coli* DH5α competent cells (TaKaRa Biotechnology, Dalian, China). Positive clones were selected and sent to Shanghai Bio-Tech for sequencing.

### Quantitative Real-Time PCR (qRT-PCR)

Gene expression studies were carried out using the SYBR Premix Ex Taq system (Takara Biotechnology, Dalian, China). All primer sequences are provided in Supporting Information Table S1. The housekeeping gene used for qPCR is *FaActin*. Total RNA was extracted from the roots and shoots of seedlings frozen by liquid nitrogen that had grown for 45 days on the four media described above using a plant total RNA extraction kit (Tiangen Biotechnology, Beijing, China). First-strand cDNAs were synthesized with the PrimeScript RT Reagent Kit with gDNA Eraser (Takara Biotechnology, Dalian, China) and used to conduct qRT-PCR analyses. The cycling protocol comprised 10 min at 95°C, then 40 cycles of 15 s at 95°C for denaturation and 1 min at 60°C for annealing and extension.The specificity was assessed by the presence of a single peak in the dissociation curve after the amplification and by size estimation of the amplified product. The comparative threshold cycle C_T_ (2^-ΔΔCT^) method was used to quantify cDNAs with amplification efficiencies equivalent to that of *FaActin*. The results shown are the average of three independent biological replicates repeated three times and technical replicates are repeated three times for each biological treatment.

### Nitrate Assimilation Enzyme Assays

The activities of nitrate redutase (NR), nitrite reductase (NiR), glutamine synthetase (GS), and NADH-glutamate synthase (NADH-GOGAT) were measured according to procedures described in Liu et al., (2017). The results shown are the average of three independent biological replicates repeated three times.

### Measurement of NO_3_^-^, NO_2_^-^, NH_4_^+^, and ^15^N Use Efficiency

The NO_3_^-^, NO_2_^-^, and NH_4_^+^ content were investigated using specific assay kits (Comin, Suzhou, China). Strawberry seedlings under each condition were cultured on MS solid media with Ca (^15^NO_3_)_2_ for 45 days. The roots and leaves were put in paper envelopes and heated at 105°C for 30 min. Then, they were dried at 80°C for 5 days. Finally, the ^15^NO_3_^-^ use efficiency was determined using a MAT-251-Stable Isotope Ratio Mass Spectrometer (Thermo Finnigan, San Jose, CA, USA) at the Institute for Application of Atomic Energy(IAAE), Chinese Academy of Agricultural Sciences (CAAS), as reported by Clarkson et al. (1996). Meanwhile, the dry weight of shoots and roots for each growth condition was measured (30 seedlings for each condition). Root length was measured using LA-S plant root analyzer (wseen, Hangzhou, China).

## Results

### Physiological and Morphological Analyses

#### Plant Growth Phenotypes

**Figure 1:**
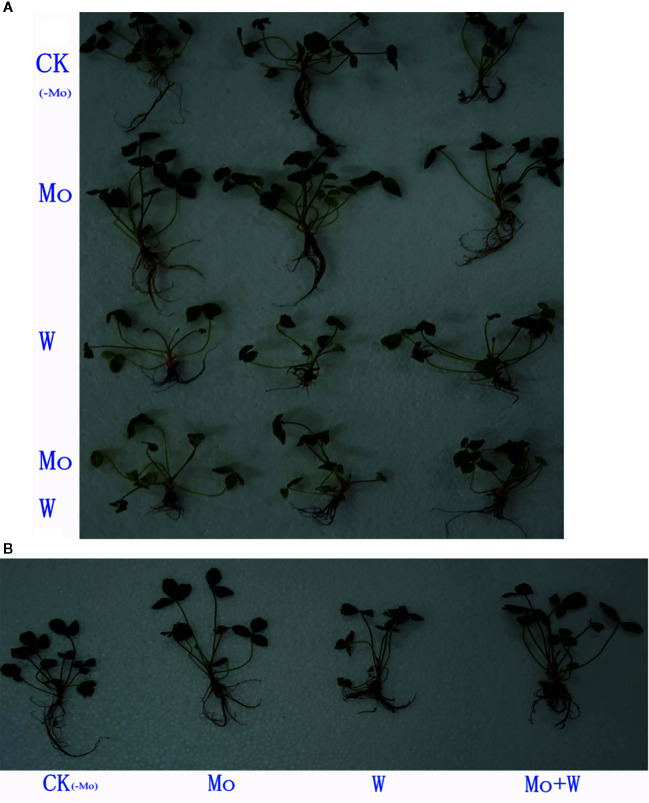
Tissue-cultured strawberry seedlings used in this experiment. Effect of solid MS culture medium with Na2MoO4 or Na2WO4 on growth status of strawberry seedlings **(A**, **B)**.

**Figure 2:**
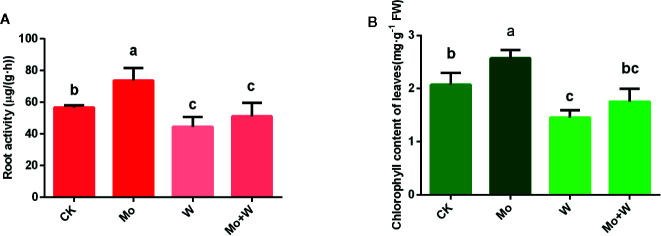
Effect of solid MS culture medium with Na_2_MoO_4_ or Na_2_WO_4_ on root activity **(A)** and chlorophyll content of leaves **(B)**. Values are means±SD. Different letter denote statistical significance according to one-way ANOVA and Duncan’s test (P <0.05).

Seedlings cultured on MS medium containing Na_2_MoO_4_ grew well and exhibited normal leaves and roots. However, seedlings cultured on the –Mo medium (CK) appeared weak, with longer roots than the Na_2_WO_4_ or the Na_2_MoO_4_+Na_2_WO_4_ condition (**Figures 1A, B**), slightly yellowed leaf blades (chlorosis) (**Figure 1B**) and low root activity (**Figure 2A**). Seedlings cultured on medium containing Na_2_WO_4_ or Na_2_MoO_4_+Na_2_WO_4_ exhibited clear differences compared with those growing on +Mo media (**Figure 1A**), and the color of the leaves is less greener as shown in **Figure 1B**. The main phenotypic differences were similar to nitrogen deficiency symptoms (i.e., reduced root and shoot growth and yellow leaves). There were no significant differences between seedlings supplied with Na_2_WO_4_ and those supplied with Na_2_MoO_4_+Na_2_WO_4_, presumably because MoO_4_^2-^ uptake was inhibited by WO_4_^2-^.

Strawberry seedlings were harvested after 45 days of culture to evaluate whether dry weight of shoots and roots were affected by treatments. As a result, there was a clear difference in the shoot dry weight between the CK and the Mo conditions (**Table 1**). The shoot dry weight of Mo treatment was 21.41% higher than the CK seedlings. Root length of the Mo condition was longer than that of the CK treatment. Root length exhibited lower in Na_2_WO_4_ and the Na_2_MoO_4_+Na_2_WO_4_ conditions than that of the CK and Mo conditions (**Table 1**). There was no significant difference between Na_2_WO_4_ and Na_2_MoO_4_+Na_2_WO_4_ conditions.

**Table 1:** The shoots and roots dry weight and root length of strawberry seedlings.

Treatment	Shoots(g)	Roots(g)	Root length(cm)
CK	3.13 ± 0.36b	2.7 ± 0.52ab	39.54 ± 12.08b
Mo	3.80 ± 0.36a	3.21 ± 0.37a	56.42 ± 12.81a
W	2.86 ± 0.13b	2.05 ± 0.63b	28.60 ± 3.69c
Mo+W	2.82 ± 0.23b	1.96 ± 0.53b	26.23 ± 5.34c

The dry weight of shoots and roots means sums of 30 strawberry seedlings for each treatment.

Values are means±SD. Different letter denote statistical significance according to one-way ANOVA and Duncan’s test (P <0.05).

#### Microstructure of Roots and Leaves

##### Root Cross-Sections

**Figure 3:**
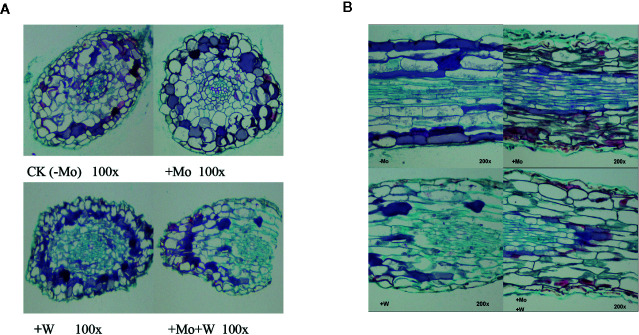
Effect of solid MS culture medium with Na_2_MoO_4_ or Na_2_WO_4_ on the microstructure of cross-section of strawberry seedling roots **(A)** and on the microstructure of longitudinal section of strawberry seedling roots **(B)**.

The apical root cells of seedlings grown on Mo-free medium (controls) were slightly deformed, loosely arranged, and exhibited enhanced cell clearance. Root tip cells of seedlings grown with sufficient Mo were arranged neatly and tightly, and the cells in the central medulla were larger (**Figure 3A**). However, in seedlings grown under +Na_2_WO_4_ conditions, the apical root cells were irregular, smaller, and more closely arranged. In addition, in seedlings grown under +Na_2_MoO_4_+Na_2_WO_4_ conditions, root tip cells were loosely arranged and exhibited abnormal shapes (**Figure 3A**).

##### Root Longitudinal Sections

Under Mo-free conditions, root tip cells exhibited an orderly arrangement and were rectangular in shape. Under standard Mo conditions, root tip cells were arranged closely (**Figure 3B**). The thickness of the longitudinal section was greater than in roots without Mo treatment (**Figure 3B**). In seedlings grown under +Na_2_WO_4_ or +Na_2_MoO_4_+Na_2_WO_4_ conditions, root tip cells were loosely arranged, irregular in shape, and uneven in size (**Figure 3B**).

##### Anatomical Structure of Leaf Tissue

**Figure 4:**
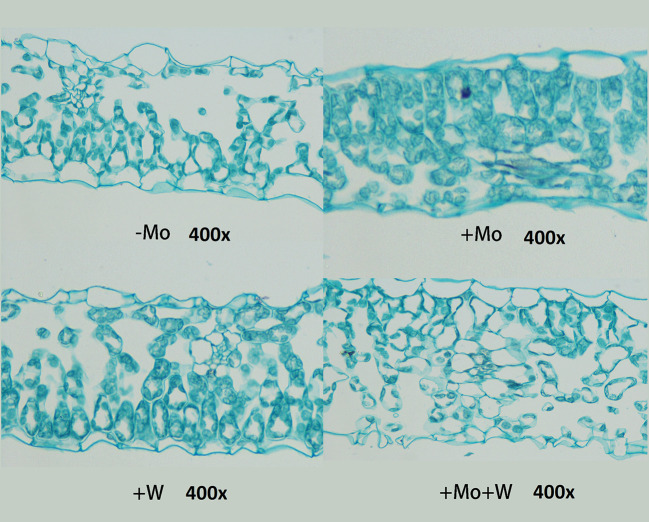
Effect of solid MS culture medium with Na_2_MoO_4_ or Na_2_WO_4_ on the microstructure of strawberry seedling leaves.

As shown in **Figure 4**, there were clear differences in leaf anatomy between the Mo-free treatment and the standard Mo treatment. The arrangement of epidermal cells in leaves was irregular, and the stomatal structure was difficult to confirm. In addition, the palisade and spongy mesophyll cells were irregular and contained large gaps. In seedlings grown on standard Mo medium, the palisade cells in normal leaves were arranged neatly and tightly with long cylindrical shapes. The palisade and spongy mesophyll cells of leaves under +Na_2_WO_4_ conditions were loosely arranged and deformed with large cell interstices.

#### Ultrastructure of Leaves

##### Leaves Cultured Without Mo

**Figure 5:**
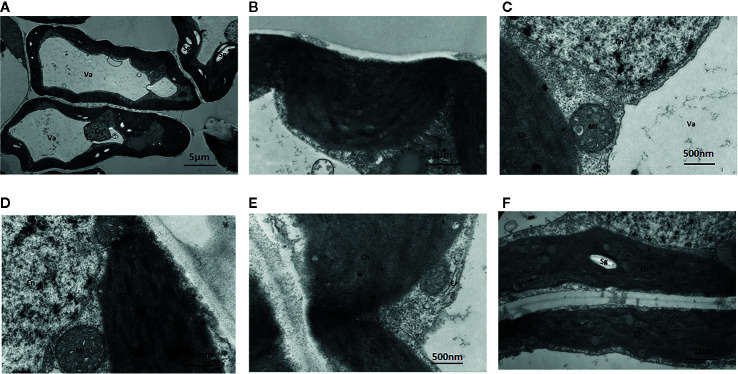
Effect of solid MS culture medium without Na_2_MoO_4_ on cell morphology and ultrastructure in leaf of strawberry seedlings. Transmission electron micrographs of leaves in strawberry seedlings examined under a JEOL-1200EX TEM TEM. **(A)** The cellular structure **(B)** The swollen chloroplast. **(C)** The membrane system is damaged and the membrane structure is unclear. **(D)** Cn, Mi, Ch is unclear **(E)** Irregular chloroplasts and Oedema of the endoplasmic reticulum **(F)** Sg in the chloroplasts. Ch, chloroplast; Cn, cell nucleus; Er, endoplasmic reticulum; Mi, mitochondria; Va, vacuole; Sg, starch grain.

Under Mo-free conditions, much of the mesophyll cell ultrastructure was abnormal, and the organelles and internal membrane system showed senescence associated with a visual loss of chlorophyll. These changes were likely caused by Mo deficiency. Some chloroplasts were deformed and swollen with unclear boundaries. Others were not well developed and had a dark appearance (**Figure 5**). Gaps between the grana were larger and contained white spaces, and the lamellae were blurred. The chloroplast and mitochondrial membranes were damaged. Additionally, the endoplasmic reticulum had swollen and shed ribosomes, likely reducing the cell’s ability to synthesize proteins.

##### Leaves Cultured With Standard Mo

**Figure 6:**
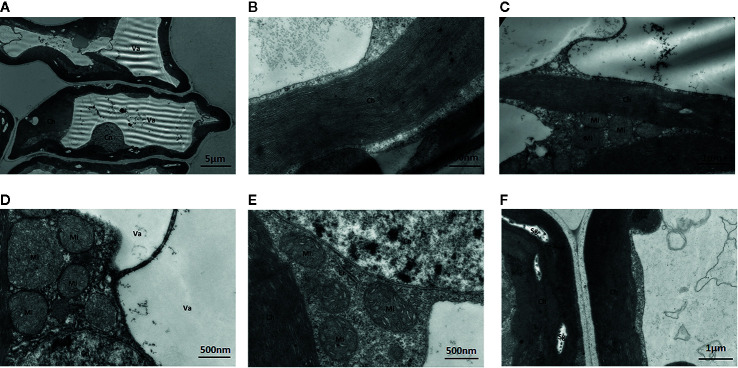
Effect of solid MS culture medium with Na_2_MoO_4_ on cell morphology and ultrastructure in leaf of strawberry seedlings. **(A)** The whole cell with organelles. **(B)** Stroma lamella and grana lamella in chloroplast. **(C)** the well developed chloroplasts. **(D)** the well-developed membrane of Mi and Cn, with mitochondrial cristae. **(E)** Er, Mi, Cn, and Ch of strawberry seedling grown with MoO_4_^2-^ **(F)** The well-developed chloroplast with several Sgs. Ch, chloroplast; Cn, cell nucleus; Er, endoplasmic reticulum; Mi, mitochondria; Va, vacuole; Sg, starch grain.

Under standard Mo conditions, mesophyll cell structure was well developed with abundant organelles and a complete internal membrane system (**Figure 6**). Chloroplasts were abundant, contained more starch granules and some osmiophilic granules, and exhibited a complete two-layer membrane structure. The granal and stromal thylakoids were regular in shape and compactly arranged (**Figure 6**). The mitochondria were well developed, and the crest structures of some mitochondria were clearly visible.

##### Leaves Cultured With Na_2_WO_4_

**Figure 7:**
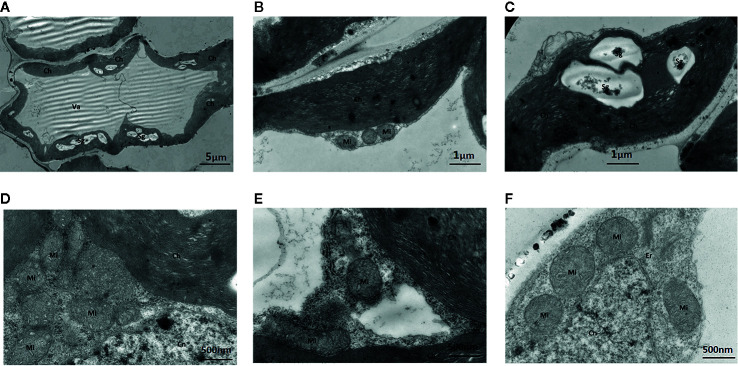
Effect of solid MS culture medium with Na_2_WO_4_ on cell morphology and ultrastructure in leaf of strawberry seedlings. **(A)** The abnormally shaped cell with organelles. **(B)** The irregular chloroplast of seeding under Na_2_WO_4_ condition. **(C)** The poorly developed chloroplast with Sgs. **(D)** Mi, Cn and Ch in the cell **(E)** Mi, Er, Cn and Ch in the cell. **(F)** Mi, Er, and Cn in the cell. Ch, chloroplast; Cn, cell nucleus; Er, endoplasmic reticulum; Mi, mitochondria; Va, vacuole; Sg, starch grain.

The addition of the Mo competitive inhibitor Na_2_WO_4_ led to severe mesophyll cell damage, a deformed arrangement of cells, and the destruction of organelles (**Figure 7**). The membrane structure was unclear and the external envelopes were incomplete. The numbers of chloroplasts and granal lamellae were significantly decreased, likely reducing the leaf’s ability to synthesize carbohydrates, resulting in lower chlorophyll content. Some chloroplasts were round with damaged lamellae and a larger number of osmiophilic particles (**Figure 7**).

##### Leaves Cultured With Na_2_WO_4_ and Na_2_MoO_4_

**Figure 8:**
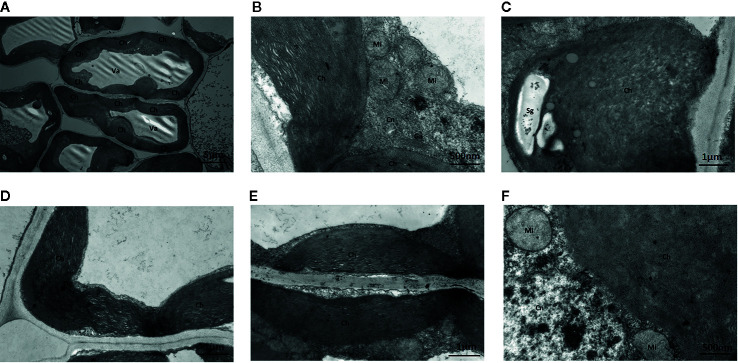
Effect of solid MS culture medium with Na_2_MoO_4_ and Na_2_WO_4_ on cell morphology and ultrastructure in leaf of strawberry seedlings. **(A)** The whole cell with organelles of seedlings grown with Na_2_MoO_4_ and Na_2_WO_4_. **(B)** Ch, Cn, Ga, Mi in the cell. **(C)** The swollen chloroplast. **(D)** The poorly developed chloroplast with osmiophilic granules deposited. **(E)** Damaged chloroplasts in the seeding cultured with inhibitor. **(F)** Mi, Cn, and Ch in the cell. Ch, chloroplast; Cn, cell nucleus; Er, endoplasmic reticulum; Ga, Golgi apparatus; Mi, mitochondria; Va, vacuole; Og, osmiophilic granules; Sg, starch grain.

Under Na_2_WO_4_+Na_2_MoO_4_ conditions, cells and chloroplasts were irregular in shape with an unclear membrane structure. Both granal and stromal lamellae were severely damaged, and the interlamellar space between the granal lamellae was larger (**Figure 8**). Chloroplasts were markedly smaller compared with those from seedlings under standard Mo conditions, indicating that chlorophyll content was reduced in strawberry seedlings under Na_2_WO_4_+Na_2_MoO_4_ treatment as a result of the loss of MoO_4_^2-^ (**Figure 8**).

Given that Mo deficiency led to reductions in spongy and palisade parenchyma, chloroplast deformation, and reduced chloroplast numbers, we next investigated the chlorophyll content of seedlings cultivated under sufficient and limited Mo conditions. Chlorophyll content was higher in seedlings grown under standard Mo conditions than in seedlings cultivated without Mo supplementation (**Figure 2B**). Chlorophyll content was also relatively lower in seedlings cultured in the Na_2_WO_4_ supplemented medium (**Figure 2B**). These results established that Mo deficiency phenotypes were similar to those of nitrogen deficiency. Therefore, phenotypes of Mo deficiency may have been wrongly identified as nitrogen deficiency in the past.

#### *FaMOT1* Sequence Analysis

A Mo transporter gene, *FaMOT1*, was cloned from a strawberry “Akihime” seedling. Its coding sequence is 1,365 bp in length, and it encodes a putative protein of 454 amino acids, 219 hydrophobic, and 235 hydrophilic. The predicted molecular weight of the FaMOT1 protein is 48,222.2 Da, and its theoretical isoelectric point is 9.14. FaMOT1 was predicted to be localized to the vacuole using SoftBerry ProtComp 9.0 (http://linux1.softberry.com/berry.phtml).

#### *MOT1* Expression in Shoots and Roots and Molybdate Concentration Assay

**Figure 9:**
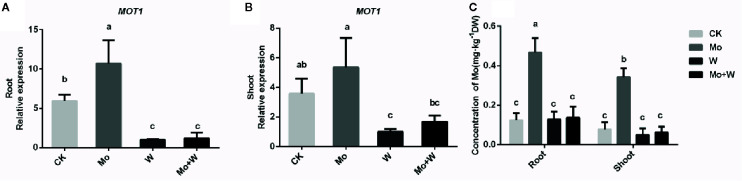
*MOT1* gene expressions in the roots **(A)**, shoots **(B)**, and molybdenum concentration of roots and shoots **(C)** of strawberry seedlings applied with treatments (CK, Mo, W, Mo+W). Data is presented as the means ± SD, n = 3. Different letters above bars denote statistical significance according to one-way ANOVA and Duncan’s test (P < 0.05).

Elemental profile analysis showed that the Mo concentration of shoots and roots was the highest in plants grown under +Mo conditions (**Figure 9C**). There were no significant differences in Mo concentration among seedlings grown on Mo-free medium and those grown with a molybdate inhibitor (Na_2_WO_4_ or Na_2_MoO_4_+Na_2_WO_4_), which may reflect endogenous Mo present in the seedlings. Mo content in leaves was higher than that of roots grown under standard Mo conditions (**Figure 9C**), suggesting that Mo is a mobile element and Na_2_MoO_4_ added to the medium was transferred from roots to shoots.

To investigate whether the expression of the *FaMOT1* gene was regulated by Mo availability, we performed quantitative RT-PCR to quantify *FaMOT1* mRNA in tissue culture seedlings grown under Mo sufficient or deficient conditions. Under standard Mo conditions, *FaMOT1* expression in shoots and roots was approximately 50% higher than that of seedlings cultivated under Mo-free conditions (**Figures 9A, B**). *FaMOT1* expression in shoots and roots was strongly suppressed by a molybdate inhibitor (under Na_2_WO_4_ or Na_2_MoO_4_+Na_2_WO_4_ conditions) (**Figures 9A, B**).

#### Expression of Genes Related to the Moco Biosynthesis Pathway

**Figure 10:**
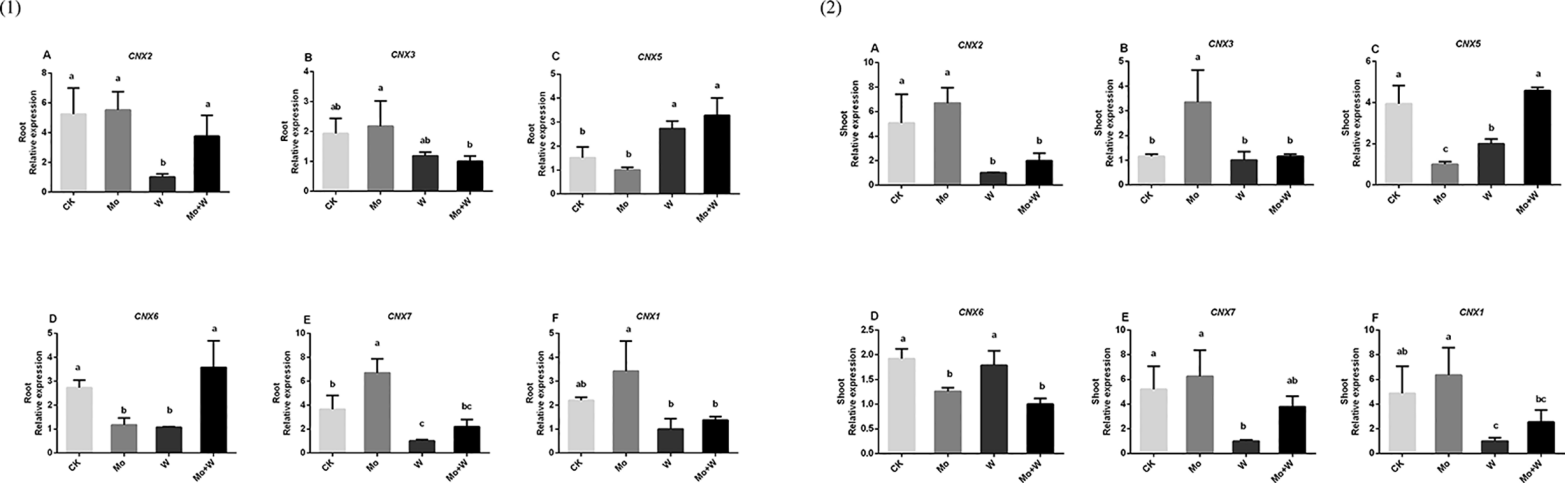
*CNX2* **(A)**, *CNX3* **(B)**, *CNX5* **(C)**, *CNX6* **(D)**, *CNX7* **(E)** and *CNX1* **(F)** gene expressions in roots (1) and *CNX2* **(A)**, *CNX3* **(B)**, *CNX5* **(C)**, *CNX6* **(D)**, *CNX7* **(E)** and *CNX1* **(F)** gene expressions in shoots (2) of strawberry seedlings applied with treatments (CK, Mo, W, Mo+W). Data is presented as the means ± SD, n = 3. Different letters above bars denote statistical significance according to one-way ANOVA and Duncan’s test (P < 0.05).

The first step in Moco synthesis is the formation of cyclic pyranopterin monophosphate (cPMP) by guanosine triphosphate (GTP) under the action of cofactor for nitrate reductase and xanthine dehydrogenase 2 (CNX2) and nitrate reductase and xanthine dehydrogenase 3 (CNX3). The expression of *CNX2* and *CNX3* genes was higher in the roots of seedlings grown on Mo sufficient medium than in the roots of seedlings grown on medium with a Mo inhibitor [**Figures 10(1)A, B**]. A similar trend was observed in the shoots [**Figures 10(2) A, B**] The second step in Moco synthesis is the conversion of cPMP to MPT, catalyzed by CNX5, CNX6, and CNX7. Both the shoots and roots of seedlings cultured under standard Mo conditions showed strongly increased *CNX7* expression [**Figures 10(1)E**, **10(2)E**]. The Mo inhibitor (Na_2_WO_4_) enhanced the expression of *CNX5* in roots and shoots [**Figures 10(1)C**, **10(2)C**]. *CNX6* was highly expressed in the roots of plants grown on Mo-free medium or medium containing Na_2_MoO_4_+Na_2_WO_4_ [**Figures 10(1)D**, **10(2)D**]. In the final biosynthetic step, Moco is formed by activating MPT in the presence of CNX1. The expression of *CNX1* was increased in plants under +Mo conditions compared with plants under –Mo or Mo inhibitor (+ Na_2_WO_4_) conditions [**Figures 10(1)F**, **10(2)F**].

#### Utilization of NO_3_^−^, NO_2_^−^ and NH_4_^+^

**Figure 11:**
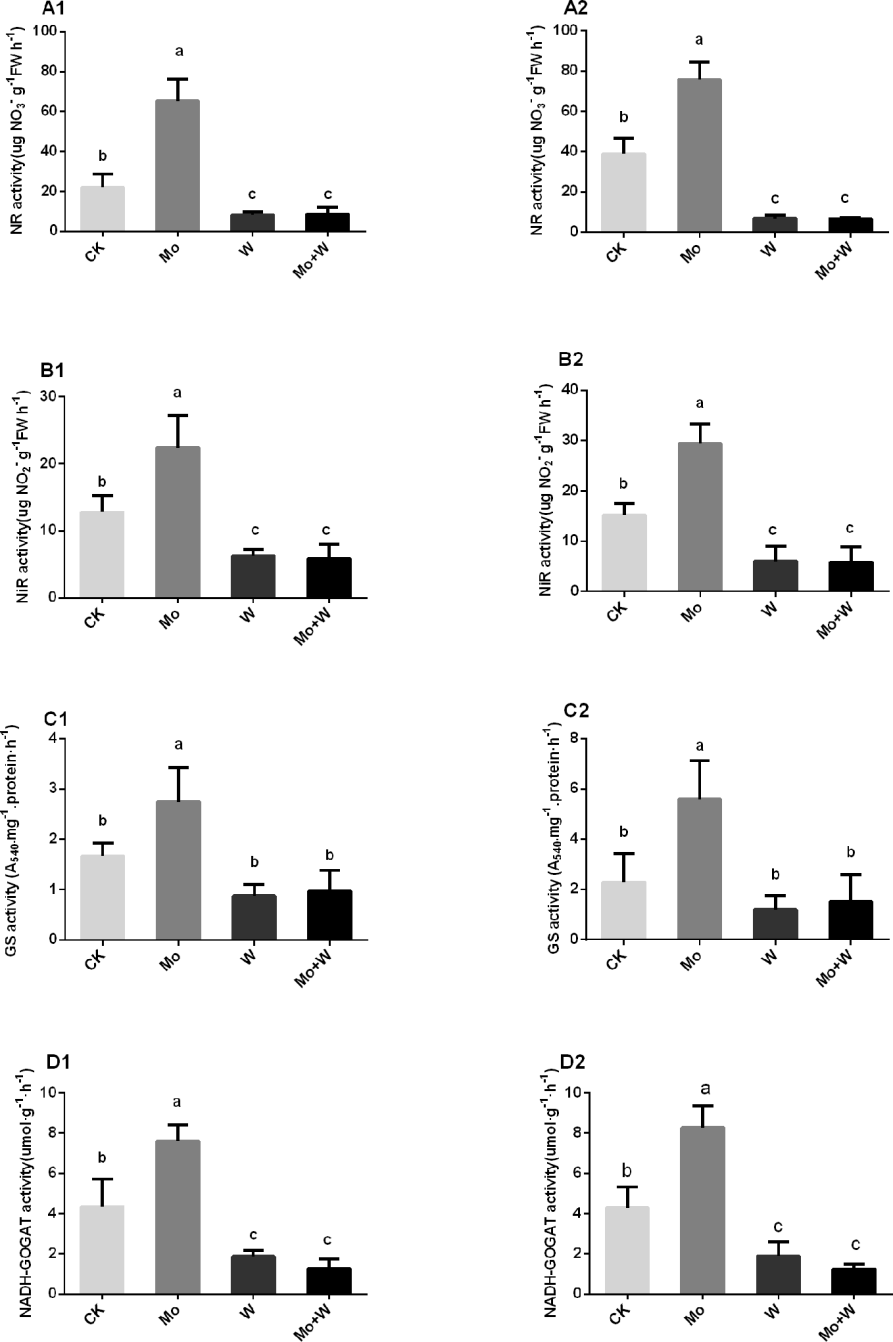
NR activities in the shoots **(A1)**, and roots **(A2)**; NiR activities in the shoots **(B1)**, and roots **(B2)**; GS activities in the shoots **(C1)**, and roots **(C2)**, and NADH-GOGAT activities in the shoots **(D1)**, and roots **(D2)** of strawberry seedlings applied with treatments (CK, Mo, W, Mo+W). Data is presented as the means ± SD, n = 3. Different letters above bars denote statistical significance according to one-way ANOVA and Duncan’s test (P < 0.05).

**Figure 12:**
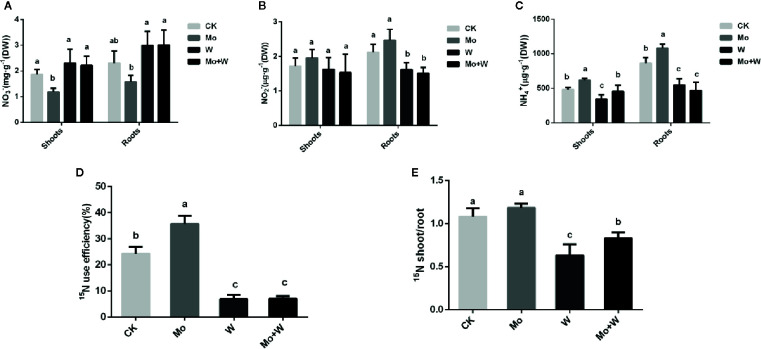
The NO_3_^-^ **(A)**, NO_2_^-^ **(B)**, NH_4_^+^ **(C)**, 15N use efficiency (%) **(D)** and 15N shoot/root ratio (E) in strawberry seedlings applied with treatments (CK, Mo, W, Mo+W). Data is presented as the means ± SD, n = 3. Different letters above bars denote statistical significance according to one-way ANOVA and Duncan’s test (P < 0.05).

The presence of the Mo inhibitor Na_2_WO_4_ impaired the activity of the Moco-dependent enzyme, NR. NR activity was therefore higher under +Mo conditions (**Figures 11A1, A2**), to the benefit of NO_3_^−^ utilization with lower NO_3_^−^ concentration in roots (**Figure 12B**). Similar trends were observed in the activities of other enzymes involved in N metabolism and assimilation, including NiR, GS, and NADH-GOGAT (**Figures 11B–D**). No significant differences of NO_3_^−^ concentration were observed in shoots among the four treatments (**Figure 12A**). However, root NO_3_^−^ concentration was higher than shoot NO_3_^−^ concentration under both –Mo and +Mo conditions (**Figure 12A**), and concentrations of NH_4_ *^+^* were higher in plants cultivated under +Mo conditions (**Figure 12C**), suggesting that more NH_4_ *^+^* was formed in order to meet the demand for amino acid synthesis.

To verify whether Mo can enhance nitrogen efficiency (NUE), ^15^N-Ca(NO_3_)_2_ was added to the MS medium. Results showed that more ^15^N-Ca(NO_3_)_2_ was translocated from roots to shoots in seedlings grown under +Mo conditions (**Figure 12E**), and as expected, ^15^N utilization rate was the highest for this treatment (**Figure 12D**). By contrast, more residual ^15^N-Ca(NO_3_)_2_ was observed in the roots of seedlings cultivated with Na_2_WO_4_, and ^15^N utilization rate was lower in the two inhibitor treatments compared with the control treatment (**Figure 12D**).

## Discussion

Mo is an essential element that is indispensable for plant growth and development. All plants require Mo for the biosynthesis of Moco, which has vital roles in nitrate assimilation, abscisic acid biosynthesis, purine degradation, and sulfite detoxification (Bittner, 2014). However, Mo deficiency phenotypes, the molecular mechanisms that regulate Mo uptake and transport, and the relationship between Mo nutrition and nitrate metabolism are poorly understood in fruit crops, particularly strawberry. A better understanding of Mo nutrition is crucial for the development of sustainable strawberry production under Mo-limited conditions.

In this study, morphological and physiological characteristics of strawberries were characterized under Mo deficient conditions using the molybdate-free medium and medium containing the molybdate analog anion tungstate. Strawberry seedlings grew well under Mo sufficient conditions, with normal green leaves and extensive root systems. By contrast, plants cultivated without Mo exhibited typical symptoms of Mo depletion, including impaired growth, yellowing leaves, and few lateral roots. These symptoms were also observed in *Lotus japonicus* grown without Mo for 45 days (Gao et al., 2016). The use of the molybdate analog tungstate also caused Mo deficiency phenotypes, which were similar to nitrogen deficiency symptoms such as leaf blade yellowing. In addition, root and shoot histology, leaf ultrastructure, leaf chlorophyll content, and the morphology and physiological function of the root system were investigated to better understand why leaf yellowing and poor root growth occur under Mo deficient conditions. Strawberry seedlings grown under standard Mo conditions showed a well-developed microstructure and ultrastructure, a large number of chloroplasts, and a higher chlorophyll content. Enhanced root activity and well-developed root systems were observed when seedlings were cultivated on a standard Mo medium. Improved root system traits included enhanced root length and surface area, larger numbers of tips, forks, and crossings, and increased root volumes, all of which were potentially beneficial to the absorption and utilization of MoO_4_^2-^ and NO_3_^-^. Improved MoO_4_^2-^ and NO_3_^-^ nutrition would presumably enhance photosynthetic capacity, providing more reducing power for nitrate reduction and overall nitrogen assimilation. Seedlings cultivated on MS medium without Mo displayed stunted root systems with decreased root activity, implying that their ability to absorb mineral elements was impaired. They also exhibited poorly developed palisade and spongy mesophyll tissue with swollen and less numerous chloroplasts, suggesting a lower chlorophyll content. Together, these results show that Mo deficiency can lead to abnormal root and leaf structure as a result of disruption in the synthesis and transport of nutrients, particularly Mo homeostasis and nitrogen metabolism. Results from the addition of the molybdate analog tungstate further confirmed this conjecture.

Mo, as molybdate, enters plant cells through specific transporters belonging to the MOT1 (Molybdate Transporter type 1) or MOT2 (Molybdate Transporter type 2) families. MOT1 family molybdate transporters were first discovered in *C. reinhardtii* and Arabidopsis (Tejada-Jiménez et al., 2007; Tomatsu et al., 2007). Knocking out AtMOT1;1 resulted in decreased accumulation of Mo in roots and shoots, and the *atmot1;1* mutant exhibited Mo deficiency symptoms when grown under limited Mo supply, indicating an essential role for AtMOT1;1 in the uptake of Mo from the soil (Tomatsu et al., 2007; Baxter et al., 2008). Huang et al. (2018) demonstrated that the rice Mo transporter OsMOT1;1 transported and controlled Mo concentrations in grain. Until now, the role of MOT1 proteins from small fruit in molybdate uptake, transport, and homeostasis has not been investigated. In this study, a putative Mo transporter with conserved characteristics of the MOT1 family, FaMOT1, was isolated from *Fragaria* × *ananassa* Duch. A phylogenetic tree showed that *FaMOT1* was closely related to *MdMOT1* from *Malus domestica* and *PpMOT1* from *Prunus persica*, both of which are also members of the Rosaceae family (**Figure S1**). In addition, the conserved domains analysis shows that the functional domain of FaMOT1 is MFS_MOT1, which is a family of molybdenate transporters (**Figure S1C**). The above results suggest that FaMOT1 belongs to the MOT1 family and may be responsible for molybdate uptake and transport in strawberry. However, a characterization of FaMOT1 requires much more work in the future. *FaMOT1* expression was higher under Mo-sufficient conditions than under Mo-limited conditions, suggesting that more MoO_4_^2-^ was taken up and translocated from roots to shoots. This is consistent with the higher Mo concentrations observed in seedlings supplied with MoO_4_^2-^. In addition, this study provides evidence once again for that the molybdate analog tungstate partially inhibited molybdate uptake and caused lower Mo concentrations, consistent with previous results in *Saccharomyces* (Tejada-Jiménez et al., 2007). Corresponding higher Mo concentrations suggest that more nitrate is assimilated in Mo-sufficient strawberry seedlings because Mo is required for NR activity during nitrate assimilation. Moco concentrations, the expression of Moco biosynthetic genes, and NR activity were therefore measured to directly test the relationship between Mo supply and nitrate reduction. Our results showed that seedlings grown in the presence of molybdate exhibited higher Mo concentrations for incorporation into Mo-requiring enzymes such as NR. As expected, nitrate reductase activity was correlated with Mo concentration. CNX2 and CNX3 are involved in the first Moco biosynthesis step, conversion of guanosine triphosphate (GTP) to cyclic pyranopterin monophosphate (cPMP). The expression levels of *CNX2* and *CNX3* were higher in cultured seedlings with MoO_4_^2-^ added in the medium. CNX5, CNX6, and CNX7 convert cPMP into molybdopterin (MPT). *CNX7*was highly expressed in the cultured seedlings with MoO_4_^2-^ in the medium. We should pay more attention to the detailed roles of CNX5, CNX6 and CNX7 in Moco biosynthesis in strawberry. In the third step, molybdate is transferred to MPT to form Moco, which is carried out by the CNX1 G-domain in a Mg^2+^ and ATP-dependent manner, thus generating molybdopterin-adenosine monophosphate (MPT-AMP). MPT-AMP serves as a substrate for the subsequent Mo insertion reaction to form Moco, which is carried out by the E-domain of CNX1. The expression of *CNX1* in the cultured seedlings cultivated with sodium MoO_4_^2-^ was higher than that of the cultured seedlings with inhibitor. The above results indicated that the synthesis reaction of Moco may be more intense in the cultured seedlings with MoO_4_^2-^ in the medium, which may be beneficial to the synthesis of molybdenum enzymes (such as NR). Next, to determine whether more nitrate was assimilated in seedlings supplied with sufficient molybdate, we assessed the activities of other nitrate metabolic enzymes and the concentrations of different nitrogen forms. The activities of other nitrate metabolic enzyme responded to Mo in a manner similar to that of NR. When Mo was added to the medium, seedling nitrate concentrations decreased and NH_4_^−^-N concentrations increased, suggesting that nitrate reduction was more intense under normal Mo supply. The higher Mo concentrations observed in roots suggested that nitrogen assimilation may be more intense in roots than in shoots. Finally, we performed a ^15^N-Ca(NO_3_)_2_ labeling experiment to determine whether more nitrate was reduced and utilized under sufficient Mo supply. As expected, more ^15^N was transferred from roots to shoots when seedlings were grown under normal Mo supply, indicating a higher ^15^N utilization rate. Therefore, more nitrate was assimilated and utilized in plants supplied with sufficient Mo.

## Conclusion

Strawberry seedlings cultured with MoO_4_^2-^ grew well and exhibited normal microstructure and ultrastructure of leaves and roots. Seedlings cultivated under Mo-deficient conditions showed yellow leaf blades, irregular chloroplasts, and unclear membrane structures.

Seedlings cultivated with MoO_4_^2-^ altered expression of genes in Moco biosynthesis (*CNX2*,*CNX3*,*CNX5*,*CNX6*,*CNX7*,*CNX1*) and exhibited higher NR activity compared to that with inhibitor Na_2_WO_4_.

Strawberry seedlings grown under Mo-deficient supply exhibited decreased ^15^NO_3_^-^ translocation and lower ^15^NO_3_^-^ use efficiency.

Strawberry seedlings cultured with Mo is beneficial to nitrate assimilation and utilization.

## Data Availability Statement

The raw data supporting the conclusions of this article will be made available by the authors, without undue reservation, to any qualified researcher.

## Author contributions

LL, FR, and YW designed the research. LL, HS, and SL performed the experiments. LL, SM, and RZ analyzed the data. LL and YW wrote the manuscript. LL and FR revised the intellectual content of this manuscript. All authors contributed to the article and approved the submitted version.

## Funding

This study was funded by the National Natural Science Foundation of China (Grant number 31801930).

## Conflict of Interest

The authors declare that the research was conducted in the absence of any commercial or financial relationships that could be construed as a potential conflict of interest.
