# Supplementary material for: Integrated Analysis of Molybdenum Nutrition and Nitrate Metabolism in Strawberry
Source: Front Plant Sci. 2020 Jul 28;11:1117. doi: 10.3389/fpls.2020.01117 (PMC7399381; doi:10.3389/fpls.2020.01117)
Supplement: Supplementary file 1 [file Table_1.docx]

| Primers’ names | Primer sequences（5’→3’） |
| --- | --- |
| *FaActin*-F | TGGGTTTGCTGGAGATGAT |
| *FaActin*-R | CAGTTAGGAGAACTGGGTGC |
| *FaMOT1*-F | CAACTCGCACAGGGATTGTCATTT |
| *FaMOT1*-R | TCCTCAACTCCACCTTCACTCAC |
| *FaCNX1*-F | CGCTTTCTTTCTGGATGGG |
| *FaCNX1*-R | GGAAGCCAGTACTCTCAGC |
| *FaCNX2*-F | CTCTGGTCCCTGCAAAGTT |
| *FaCNX2*-R | GTCAGCTCCACAAAATCGC |
| *FaCNX3*-F | AATCTCATCCCGCTATGCCA |
| *FaCNX3*-R | CCCACCAGCCTTACTCTCAA |
| *FaCNX5*-F | CAAGAGCCCTTACGCACATC |
| *FaCNX5*-R | CCCTTCAAATCCGAGTGCAG |
| *FaCNX6*-F | GCAACGTTTTCTGGTACCAC |
| *FaCNX6*-R | CGATGAACAGATGAGACCGC |
| *FaCNX7*-F | TCACTGAGATGCCACTGGAG |
| *FaCNX7*-R | TGGAGGTATGATGGCCAACT |

Primer sequences used in qRT-PCR analyses
