# Supplementary figures and images for: Integrated Analysis of Molybdenum Nutrition and Nitrate Metabolism in Strawberry
Source: Front Plant Sci. 2020 Jul 28;11:1117. doi: 10.3389/fpls.2020.01117 (PMC7399381; doi:10.3389/fpls.2020.01117)

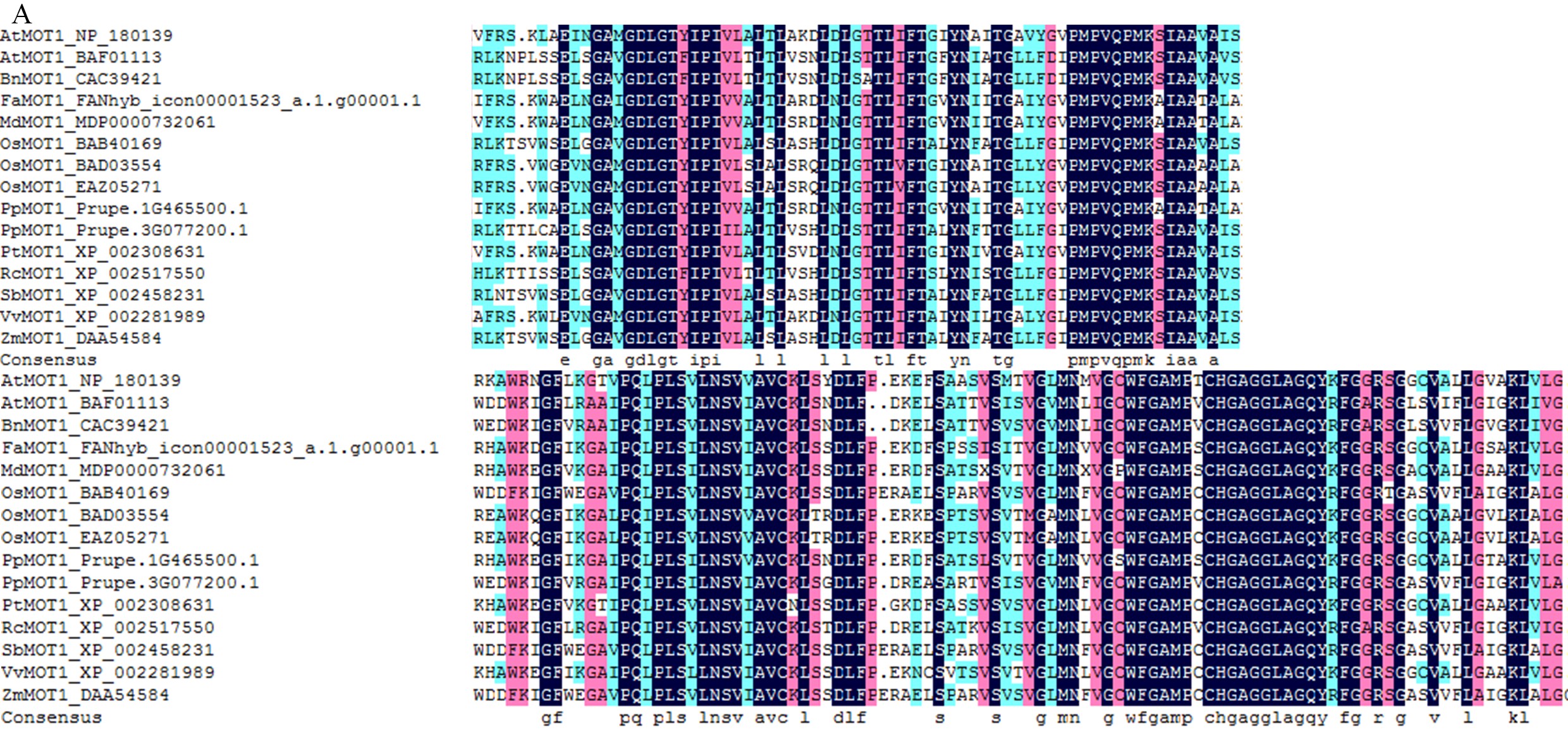

Supplement: Supplementary file 2 [file Image_1.jpg]

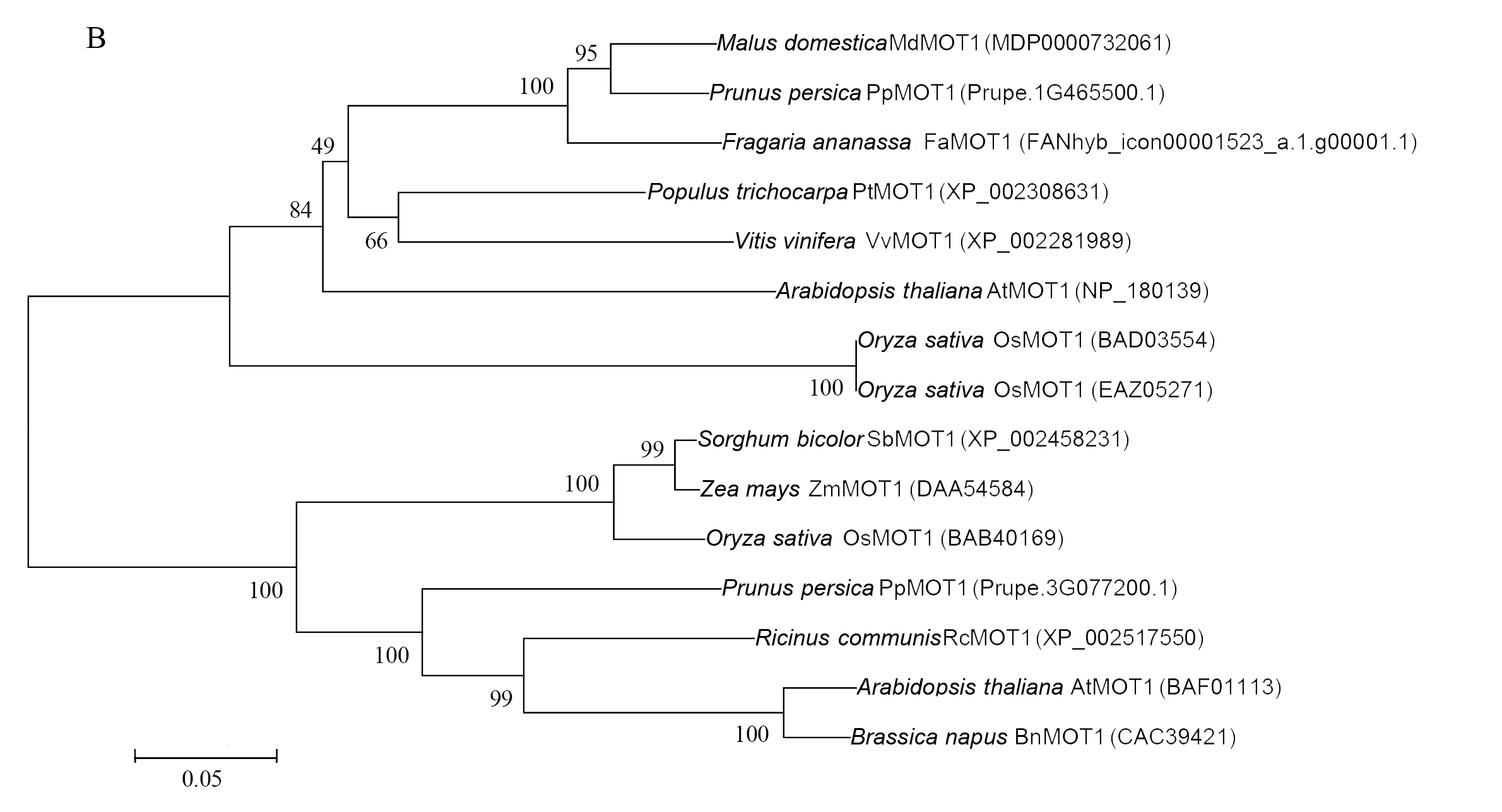

Supplement: Supplementary file 3 [file Image_2.tif]

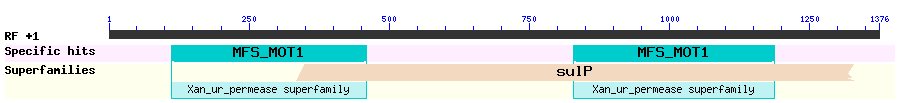

Supplement: Supplementary file 4 [file Image_3.tif]
